# Heterogeneity assessment of vaccine‐induced effects using point‐of‐care surrogate neutralization test for severe acute respiratory syndrome coronavirus 2

**DOI:** 10.1002/jcla.24545

**Published:** 2022-06-09

**Authors:** Yoshiyuki Watanabe, Ikuro Matsuba, Karin Watanabe, Tomoyuki Kunishima, Yukako Takechi, Tetsuo Takuma, Yasushi Araki, Nobuo Hirotsu, Hiroyuki Sakai, Ritsuko Oikawa, Hiroki Danno, Masakazu Fukuda, Seiji Futagami, Kota Wada, Hiroyuki Yamamoto, Fumio Itoh, Ichiro Oda, Yutaka Hatori, Hisakazu Degawa

**Affiliations:** ^1^ Kawasaki Physicians Association Kawasaki Japan; ^2^ Department of Internal Medicine Kawasaki Rinko General Hospital Kawasaki Japan; ^3^ Department of Internal Medicine, Division of Gastroenterology St. Marianna University School of Medicine Kawasaki Japan; ^4^ Department of Internal Medicine, Division of Gastroenterology Nippon Medical School Tokyo Japan; ^5^ Department of Otorhinolaryngology Toho University Omori Medical Center Japan; ^6^ Knowledge palette co. Ltd. Kawasaki Japan; ^7^ Department of Bioinformatics St. Marianna University Graduate School of Medicine Kanagawa Japan

**Keywords:** COVID‐19, neutralizing activity, POCT, SARS‐CoV‐2, sVNT

## Abstract

**Introduction:**

Coronavirus disease (COVID‐19) caused by severe acute respiratory syndrome coronavirus 2 (SARS‐CoV‐2) has become a global pandemic even after vaccination. We aimed to identify immunological heterogeneity over time in vaccinated healthcare workers using neutralization antibodies and neutralizing activity tests.

**Methods:**

Serum samples were collected from 214 healthcare workers before vaccination (pre) and on days 22, 90, and 180 after receiving the first dose of BNT162b2 vaccine (day 0). Neutralization antibody (NAb, SARS‐CoV‐2 S‐RBD IgM/IgG) titers and two kinds of surrogate virus neutralization tests (sVNTs) were analyzed (UMIN000043851).

**Results:**

The NAb (SARS‐CoV‐2 S‐RBD IgG) titer peaked on day 90 after vaccination (30,808.0 μg/ml ± 35,211; *p* < 0.0001) and declined on day 180 (11,678.0 μg/ml ± 33,770.0; *p* < 0.0001). The neutralizing activity also peaked on day 90 and declined with larger individual differences than those of IgG titer on day 180 (88.9% ± 15.0%, 64.8% ± 23.7%, *p* < 0.0001). We also found that the results of POCT‐sVNT (immunochromatography) were highly correlated with those of conventional sVNT (ELISA).

**Conclusions:**

Neutralizing activity is the gold standard for vaccine efficacy evaluation. Our results using conventional sVNT showed large individual differences in neutralizing activity reduction on day 180 (64.8% ± 23.7%), suggesting an association with the difference in vaccine efficacy. POCT‐sVNT is rapid and user‐friendly; it might be used for triage in homes, isolation facilities, and event venues without restrictions on the medical testing environment.

## Graphical abstract

Four points of NAbs antibody assay and sVNT. A: NAbs（SARS‐CoV‐2 S‐RBD IgG / IgM）measurement before and after vaccine on Day 22, Day 90, Day 180. The data presented represent the log of the neutralization titer for NAbs. The P values presented in A was calculated using unpaired two‐tailed Students t‐tests. B: sVNT measurement before and on Day 22, Day 90, Day 180 after the first vaccination. The dotted lines represent the cutoff at 30% inhibition. The P values presented in A was calculated from unpaired two‐tailed Students t‐tests.

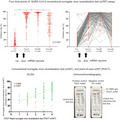

## 1 INTRODUCTION

COVID‐19 is widespread throughout the world, with a global cumulative caseload of over 100 million in early 2021; just 6 months later, the caseload increased to 200 million. Eight months later, on April 17, 2022, the number of COVID‐19 cases worldwide exceeded 500 million.^1^, ^2^ The persistent spread of infection worldwide is accelerating due to mutant strains such as the delta and omicron variants.^3^, ^4^ In the future, it is hoped that SARS‐CoV‐2 infection prevention measures such as coronavirus vaccination, the development of therapeutic agents for COVID‐19, and advances in testing methods will limit and control the spread of infection; however not yet been developed completely. We targeted 214 healthcare workers belonging to private medical institutions in Kawasaki City. We collected blood before administering the first dose of COVID‐19 BNT162b2 mRNA vaccine and on days 22, 90, and 180 after administering the first dose of the vaccine (day 0). A symptom investigation was also carried out on participants through a questionnaire. As virus neutralization tests (VNT) are strongly predictive of the degree of immune protection against SARS‐CoV‐2 infection (neutralizing activity), it might be important to understand the effect of the SARS‐CoV‐2 variant on recognition by VNT in convalescent and vaccinated individuals. Thus, we specifically analyzed the correlation between conventional surrogate virus neutralization test (sVNT) and point‐of‐care surrogate neutralization test (POCT‐sVNT) to explore the potential of POCT‐sVNTs.

## 2 MATERIAL AND METHODS

### 2.1 Healthcare workers enrolled in clinical trials

This clinical study was performed on healthcare workers at clinics and hospitals affiliated with the Kawasaki Physicians Association for 6 months (April 10, 2021, to November 10, 2021). The study was a prospective observational study, carried out using the opt‐in method, and was approved by the ethics committee of the Kanagawa Prefecture Medical Association (approval code: #R3‐0311). Subgroups, such as age and gender groups, were not defined. We decided on two factors as exclusion criteria (recent COVID‐19 vaccination and disagreement). Informed consent for the questionnaire and the blood test was obtained from all participants. Details of our study design and results have been uploaded to the University hospital Medical Information Network (UMIN) Center Clinical Trials Registry (registration number: UMIN000043851, scientific title: Short‐term safety of the BNT162b2 mRNA COVID‐19 vaccine) website. Enrollment specialists reviewed the inclusion and exclusion criteria with each potential patient to determine eligibility.

Serum samples were collected at the following time points from 214 healthcare workers who received two doses of the mRNA vaccine: prevaccination (1 day before the first vaccine) and day 22, day 90, and day 180 after the first dose of the vaccine. A questionnaire survey was completed by all of the 214 medical staff who participated in this clinical trial. The questionnaires were administered at the following time points: day 23–25, day 91–93, and day 181–183 after the first dose of the vaccine. The questionnaire contained questions regarding (1) Age and gender; (2) Injection side reaction symptoms (tears, cough, chest pain, tachycardia, chest tightness, pharyngeal strangulation, oral itching, arrhythmia, abdominal pain, tunnel vision, shortness of breath, anxiety, nasal congestion, nasal itching, vomiting, nasal discharge, sore throat, rash, itching, and dizzyness, diarrhea, fever, headache, fatigue, muscle pain); (3) smoking status; (4) habitual alcohol intake; and (5) blood type.

### 2.2 Neutralization antibodies (NAbs) measurement

Each protein (Anti‐SARS‐CoV‐2 S‐RBD protein Human IgM/IgG) was coated onto microplates (Anti‐SARS‐CoV‐2 S‐RBD protein Human IgM/IgG ELISA kit, Proteintech Group Inc.). A sufficient number of microwell strips were placed in a holder to run the controls and samples. A 100 μl each of standard and 1:100 diluted samples were added to the microwells and incubated for 30 min. Then, 100 μl of the 1x HRP‐conjugated anti‐human IgG/IgM secondary antibody was added into the microwells and incubated for 30 min. Each well was washed 4 times by dispensing 350 μl of diluted wash solution into each well, and 100 μl of the substrate was added into the microwells and incubated for 10 min. A 100 μl of the stop solution was then added to each of the microwells. The plate was read at 450 and 620 nm immediately after adding the stop solution. The best fit standard curve was determined by regression analysis using the four‐parameter logistic curve fit (4‐PL).^5^

### 2.3 Surrogate Virus Neutralization Test (sVNT) assay

The ELISA plate was precoated with ACE2 protein (SARS‐CoV‐2 Anti‐RBD Antibody Profiling Kit, Medical & Biological Laboratories Co. Ltd.). A 100 μl of positive control and samples were added to the primary reaction microplate wells. Then, 100 μl of RBD (receptor‐binding domain) reaction solution was added to the primary reaction microplate wells. After incubating for 30, 100 μl of the solution from the primary reaction microplate wells was added to the ACE2 coated microplate wells. After incubating, 100 μl of the conjugate solution was added. After incubating for 30 min, the wells were rewashed, and 100 μl of substrate solution was added for 15 min. A 100 μl of stop solution was added, then absorbance at 450 nm was measured. The inhibition rate of each sample was calculated using the following formula:

$$\text{Inhibition rate}\;\left(\%\right)=\left(1-\left(O.D.\;\text{value of sample}\right)/\left(O.D.\;\text{value of blank}\right)\right)\times 100.$$

### 2.4 POCT‐ sVNT assay

COVID‐19 S1 RBD IgG/neutralizing Ab Test is a rapid chromatographic immunoassay for the qualitative detection of S1 RBD IgG/neutralizing antibodies to SARS‐CoV‐2 in whole human blood, serum, or plasma to evaluate individual immunity after infection or vaccination (The RapiSure COVID‐19 S1 RBD IgG/neutralizing Ab Test, BioFront, Seoul, Korea). We added 25 μl of serum to each specimen well of the test device and 60 μl of the buffer separately and waited for the colored lines to appear. The test result was read at 10–15 min. When two colored bands appear on the membrane, the COVID‐19 S1 RBD IgG test results are said to be positive. One band appears in the control region (C), and another in the test region (T). When the Neutralizing Ab test is positive, the colored line in the control line region (C) changes from Blue to Red, and one colored line appears in the reference line region (R). The intensity of the colored line in the test line region (T) is weaker than the reference line in the reference region (R) or shows no apparent colored band. The result of the Neutralizing Ab test is said to be negative when the intensity of the colored line in the test line region (T) is stronger than that of the reference line in the reference region (R) (Figure 3C). Each researcher judged the concentration of the test line region (T) on a five‐point scale.

### 2.5 Statistical analysis

Questionnaire results were calculated using the unpaired t‐test. The NAbs titer was visualized using PRISM for Windows (v.7.0; GraphPad Software, San Diego, CA, USA). All statistical analyses were performed using SPSS for Windows (v.12.0; SPSS, Inc., Chicago, IL, USA) and PRISM for Windows (v.7.0; GraphPad Software, San Diego, CA, USA). No adjustment of multiple comparisons was made. All reported *p* values were two‐sided, and *p* < 0.05 was considered significant.

Correlations between NAbs and sVNT were analyzed using Pearson correlation coefficients. The Spearman's rank correlation coefficient was used to analyze correlations between NAbs, sVNT, and POCT‐sVNT. All data presented were derived from two independent experiments.

## 3 RESULTS

### 3.1 Clinical information and samples collection

There were significant differences in gender (*p* < 0.0001) and age (Male 50.2 + 15.7, Female, 44.6 + 13.5, *p* = 0.02) owing to the inclusion of clinical nurses (Table 1).

**TABLE 1:** Questionare of any symptoms after 1st and 2nd mRNA vaccination

Age	Total	*n* = 215	45.9 + 14.2
	Male	*n* = 50	50.2 + 15.7
	Female	*n* = 165	44.6 + 13.5
Symptoms	*Post 1st vaccination*		Age
	Presence	*n* = 145	43.9 + 13.6
	Absence	*n* = 70	49.9 + 14.6
	*Post 2nd vaccination*		
	Presence	*n* = 181	45.3 + 13.8
	Absence	*n* = 34	48.9 + 16.3

### 3.2 NAbs measurement of anti‐SARS‐CoV‐2 S‐RBD protein Human IgG/IgM

**FIGURE 1:**
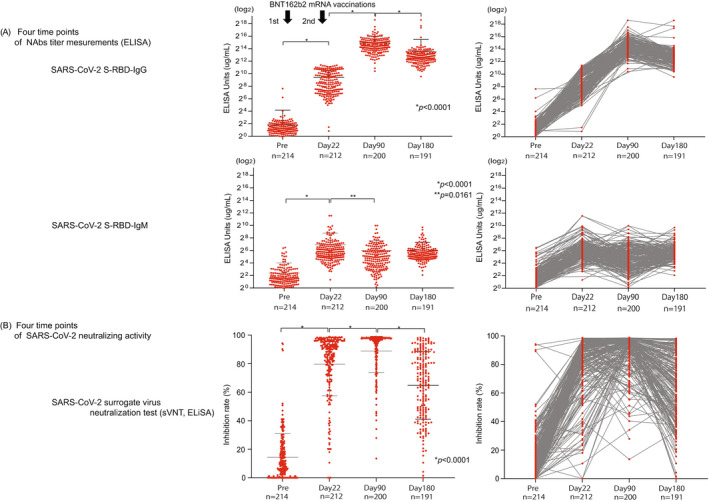
Four points of NAbs antibody assay and sVNT. (A) NAbs (SARS‐CoV‐2 S‐RBD IgG/IgM) measurement before and after vaccine on day 22, day 90, day 180. The data presented represent the log of the neutralization titer for NAbs. The *p* values presented in A was calculated using unpaired two‐tailed Student's t‐tests. (B) sVNT measurement before and on day 22, day 90, day 180 after the first vaccination. The dotted lines represent the cutoff at 30% inhibition. The *p* values presented in A were calculated from unpaired two‐tailed Student's t‐tests

**FIGURE 2:**
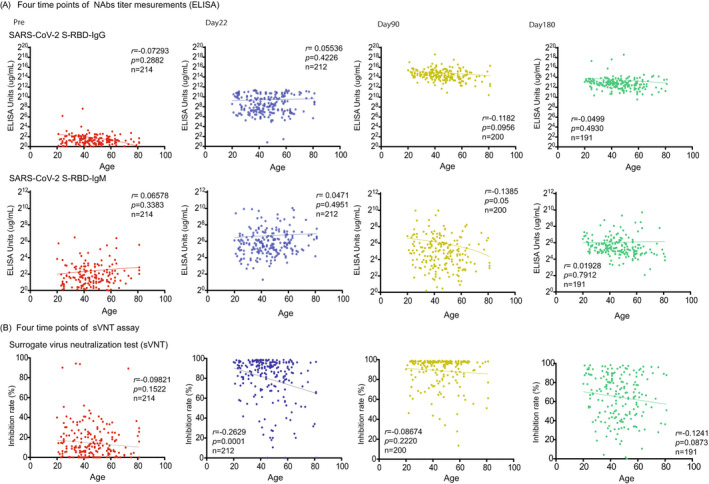
Age correlation of the NAbs antibody titer and sVNT assay in before and after vaccination. (A) The correlation between the two types of NAb titers (SARS‐CoV‐2 S‐RBD IgG/IgM) and age was investigated for each time point before and after vaccination (prevaccine: red plots, day 22: blue plots, day 90: yellow plots, day 180: green plots), but there was no clear correlation (or inverse correlation) found with age in any phase. (B) In the sVNT assay, anti‐SARS‐CoV‐2–neutralizing antibodies block HRP‐conjugated RBD protein from binding to the hACE2 protein precoated on an ELISA plate. The inhibition rate by the sVNT assay (ELISA method) correlates with age at each time point before and after the vaccination showed a weak negative correlation (*r* = −0.26, *p* = 0.0001) on day 22. Correlation and linear regression analyses were performed with GraphPad Prism 9 using Pearson's correlation coefficients

The SARS‐CoV‐2 S1 RBD IgG titer peaked on day 90 after vaccination (30,808.0 μg/ml ± 35,211; *p* < 0.0001) and then declined on day 180 (11,678.0 μg/ml ± 33,770.0; *p* < 0.0001) (Figure 1A). The titer of SARS‐CoV‐2 S1 RBD IgM was significantly elevated compared with prevaccination (1 day before the first vaccine) and significantly decreased on day 90 (Pre, 5.3 μg/ml ± 10.8, day 22, 121.7 μg/ml + 314.4; *p* < 0.0001, day 90, 64.3 μg/ml ± 124.3; *p* < 0.02) (Figure 1A). We also confirmed the NAbs titer (SARS‐CoV‐2 S1 RBD IgG/IgM) and age correlation at all time points (pre, day 22, day 90, day 180), but we found no significant correlation (pre; *n* = 214; *r* = 0.07 / *r* = 0.07; *p* = 0.29 / *p* = 0.33, day 22; *n* = 212; *r* = 0.06 / *r* = 0.05; *p* = 0.42 / *p* = 0.50, day 90; *n* = 200; *r* = 0.14 / *r* = 0.14; *p* = 0.10 / *p* = 0.05, day 180; *n* = 191; *r* = 0.49 / *r* = 0.02; *p* = 0.49 / *p* = 0.79) (Figure 2A).

### 3.3 Conventional surrogate virus neutralization test (sVNT) assay

From the conventional sVNT, we found that the average inhibition rate was raised on day 22 (Pre; 14.4% + 16.5, day 22; 79.7% + 22.3, *p* < 0.0001). Moreover, the inhibition rate further increased significantly on day 90 (day 90; 88.9% + 15.0, *p* < 0.0001); however, it then decreased with high variability on day 180 (day 180; 64.8% + 23.7, *p* < 0.0001) (Figure 1B). We also confirmed the results of sVNT and age correlation at all time points (pre, days 22, 90, and 180); however, they showed no significant correlation (pre; *n* = 214; *r* = 0.10; *p* = 0.15, day 22; *n* = 212; *r* = 0.26; *p* = 0.0001, day 90; *n* = 200; *r* = 0.09; *p* = 0.22, day 180; *n* = 191; *r* = 0.12; *p* = 0.09) (Figure 2B).

**FIGURE 3:**
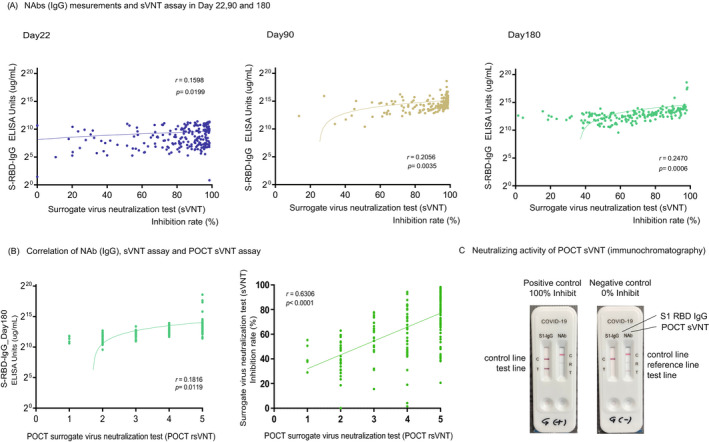
Correlation of the SARS‐CoV‐2 NAbs titer vs sVNT assays, sVNT assays vs POCT‐sVNT assays. (A) The NAbs titer (SARS‐CoV‐2 S‐RBD IgG) and sVNT (ELISA method) had a weak correlation in all phases on days 22, 90, and 180. Correlation and linear regression analyses were performed in GraphPad Prism 9 using Pearson's correlation coefficients. (B) The NAbs titer (SARS‐CoV‐2 S‐RBD IgG) had a weak correlation in all phases on days 22, 90, and 180, but sVNT assays and POCT assays had a high correlation. Correlation and linear regression analyses were performed in GraphPad Prism 9 using Spearman's rank correlation coefficients. (C) Example positive and negative results of POCT‐sVNT assays

sVNT showed a weak correlation (*r* = 0.18, *p* = 0.01) with Nab (IgG) on day 180, but the degree of decrease varied strongly from case to case between days 90 and 180 (88.9% ± 15.0%, 64.8% ± 23.7%, *p* < 0.0001) in sVNT (Figure 3A).

### 3.4 POCT surrogate virus neutralization test (POCT‐sVNT) assay

**FIGURE 4:**
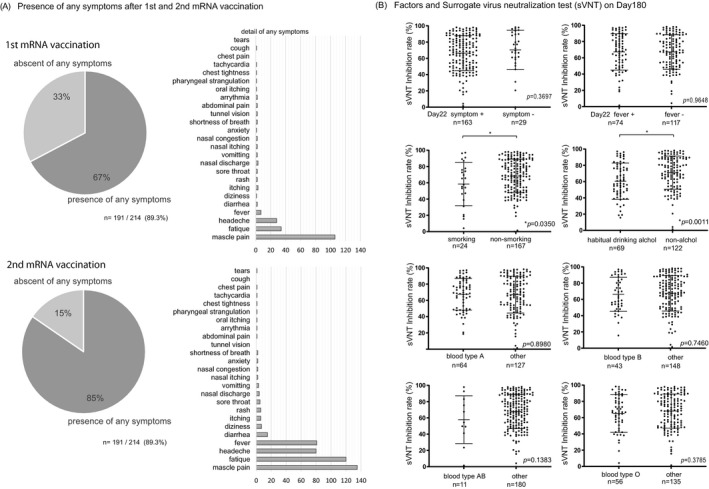
Symptoms and reactions after 1st and 2nd vaccination, and habitual factors. (A) The presence or absence of symptoms after the first and second coronavirus vaccination is shown in a Venn diagram and bar graph. Symptoms were more frequent after the second vaccination; myalgia, malaise, headache, and fever were the most common. (B) Significant differences were found only in smoking and drinking alcohol for cases with decreased neutralization on day 180. The *p* values presented in A were calculated using unpaired two‐tailed Student's t‐tests

Two independent researchers visually confirmed and quantified the density of the test band and came to a consensus. Examples of the positive and negative results for the control group are shown in Figure 3C. The inhibition rates of conventional sVNT and POCT‐sVNT were highly correlated (*r* = 0.63, *p* < 0.00001) (Figure 3B). Results showed no significant differences in age and adverse reactions on day 180, but there were significant differences in smoking/nonsmoking and habitual alcohol/nonalcohol intake (*p* = 0.0350, *p* = 0.0011) (Figure 4B).

### 3.5 Questionnaire

There were no significant differences in age for appearance of symptoms after receiving the first dose of the vaccine (presence, *n* = 144, 43.9 + 13.6: absence, *n* = 70, 49.9 + 14.6), and after receiving the second dose of the vaccine (presence, *n* = 181, 45.3 + 13.8: absence, *n* = 33, 48.9 + 16.3); however, the percentage of people with symptoms was higher after the second dose (1st 67%, 2nd 85%) (Figure 4A, Table 1). Most participants complained of muscle pain, fatigue, headache, and fever after the first and second doses (Figure 4A).

## 4 DISCUSSION

We inferred that changes in various symptoms after coronavirus mRNA vaccination and various individual factors, such as smoking history, alcohol consumption, age, and sex, might be associated with a vaccine effect that decreases over time (i.e., reduced neutralizing activity). NAbs were measured by sVNT and POCT‐sVNT over time. Results showed no significant differences in age and adverse reactions after vaccination, but there were significant differences in smoking/nonsmoking and habitual alcohol/nonalcohol intake (*p* = 0.0350, *p* = 0.0011) (Figure 4B). Furthermore, for the neutralization capacity analysis, the SARS‐CoV‐2 spike receptor‐binding domain (RBD) was an important site for establishing SARS‐CoV‐2 infection through the human angiotensin‐converting enzyme 2 (ACE2) receptor. The inoculated coronavirus mRNA vaccine sets this spike protein as a target; hence, the SARS‐CoV‐2 S1 RBD IgM and IgG antibody titers were analyzed as the neutralizing antibodies (NAbs) against the virus S1 RBD.^6^, ^7^, ^8^ When compared to SARS‐CoV‐2 S1 RBD IgM levels prior to the first vaccine dose (Pre, 5.3 μg/ml ± 10.8), those on day 22 showed a gradual increase (day 22, 121.7 μg/ml +314.4; *p* < 0.0001), followed by a decrease on day 90 (day 90, 64.3 μg/ml ± 124.3; *p* < 0.02), similar to the results in previous reports on COVID‐19 infection (Figure 1A).^9^, ^10^, ^11^ Furthermore, when compared to SARS‐CoV‐2 S1 RBD IgG levels prior to the first vaccine dose (Pre, 3.6 μg/ml ± 14.5), those on day 22 showed a significant increase (day22, 679.7 μg/ml ± 702.4; *p* < 0.0001) followed by the rise on day 90 (day 90, 30,808.0 μg/ml ± 35,211; *p* < 0.0001), and a decrease on day 180 (day 180, 11,678.0 μg/ml ± 33,770.0; *p* < 0.0001), though the value was higher than that on day 22 (Figure 1A).

IgG increases follow IgM increases in common viral infections. However, this pattern does not always apply to patients with COVID‐19, especially immunocompromised patients with long‐term PCR positivity and those with negative antibodies approximately 2 months after infection and healing.^12^, ^13^, ^14^ Regarding changes in IgG and IgM antibody titers, there are cases in which IgG does not increase after IgM. Instead, IgG increases first, and IgM hardly increases^15^; we thought this was similar to our observations in this study following coronavirus vaccination.^16^, ^17^

Recent international reports show that an increasing number of people are infected with the Omicron variant in Japan, despite receiving a second dose of COVID‐19 vaccination. Thus, the Omicron variant has been regarded as a variant of concern (VOC).^18^ Compared with the alpha strain, the Omicron variant has approximately 30 mutations, three deletions, and one insertion in the spike region. Of these, approximately 15 mutations are present in the RBD (residues 319–541), which may increase the affinity of SARS‐CoV‐2 for the human receptor ACE2.^19^ However, the antigenic escape ability of vaccination is determined by a combination of mutations and deletions in the virus; hence, it may not always respond to viruses with defects. However, a third dose of COVID‐19 vaccination could somewhat restore its effect on the Omicron variant.^20^

There have been increasing calls for an early third‐dose booster vaccination given the current situation of insufficient “coronavirus recognizability and infection control ability” expected from vaccination; however, vaccinating all people at the same time is unrealistic. We also conducted neutralization tests (binding inhibitory activity) and measured the NAbs antibody titer to understand “virus recognizability and infection control ability.”

The conventional virus neutralization test (cVNT) is the gold standard for viral antibody testing as a neutralization test for evaluating the inhibitory binding activity of the SARS‐CoV‐2 spike protein RBD to human receptor ACE2. However, the SARS‐CoV‐2 virus can only be investigated in a Biosafety Level 3 (BSL3) environment, a limited research environment requiring BSL3 experiment staff. Meanwhile, the surrogate virus neutralization test (sVNT), which evaluates the binding inhibition of spike proteins and ACE2 proteins on a plate without using the virus itself, can be conducted in a standard laboratory environment using the ELISA method.^20^, ^21^, ^22^, ^23^, ^24^ Although analyses can be performed with sVNT even at research institutions and relatively large medical institutions, it is not easy to use in the current situation, where point‐of‐care tests are needed to enable analysis at outpatient clinics and hospital wards, and in airports, workplaces, transportation facilities, hotels, and event venues, owing to the situation of the rapid spread of infection. This study introduced a simple, quick binding inhibition kit by immunochromatography rather than the conventional ELISA method for sVNT. There are high expectations for its use as a point‐of‐care test; however, these have not yet been investigated in clinical trials.^25^ This fast and simple kit can be stored between 4–30°C for 6 months. Additionally, NAbs antibody titer (SARS‐CoV‐2 S1 RBD IgG) measurements and POCT‐sVNT analysis can simultaneously be visually determined in approximately 10–15 min by dropping serum (or whole blood) into the two titration holes at the end of the kit. In this study, we conducted sVNT analysis using the ELISA method and POCT‐sVNT analysis using the immunochromatography method.

In our investigation, sVNT analysis that used the ELISA method when compared to that prior to vaccination (Pre, 14.4% ± 16.5%) increased on day 22 (day 22, 79.7% ± 22.3%; *p* < 0.0001) and showed a very high inhibitory capacity on day 90 (day 90, 88.9% ± 15.0%; *p* < 0.0001), after which it decreased on day 180 (day 180, 64.8% ± 23.7%; *p* < 0.0001) (Figure 1B). The extent of the decrease in sVNT on day 180 varied from case to case. Some cases still had strong binding inhibitory activity, and in other cases, it decreased to the same level as before vaccination (Figure 1B). The individual differences among these cases showed a low correlation with the SARS‐CoV‐2 S1 RBD IgG antibody titer (day 22, *r* = 0.16, *p* = 0.02: day 90: *r* = 0.21, *p* = 0.004, day 180: *r* = 0.25, *p* = 0.0006) (Figure 3A), but no correlation was found by age and gender (Figure 2B). Furthermore, no significant differences were found in the presence or absence of adverse reactions or blood type. However, significant differences in smoking (smoking, 58.54% + 26.7: nonsmoking, 68.48% + 20.6, *p* = 0.04) and habitual alcohol intake (alcohol, 60.50% + 22.3: nonalcohol, 71.04% + 20.4, *p* = 0.001) were found; it was inferred that these two factors affected the fast binding inhibitory activity following vaccination (Figure 3B). Furthermore, a comparative investigation between sVNT and POCT‐sVNT measurement showed a correlation on day 180 (*r* = 0.63, *p* < 0.00001) (Figure 3B).

NAbs antibody titer measurement is thought to be effective in determining the “ability to recognize the virus” by antibodies produced from human‐acquired immunity after regular vaccination; however, it has been reported that the number of antibody‐producing cells produced by the novel COVID‐19 vaccine (mRNA vaccine) gradually decreases and the antibody concentration drops to approximately one‐fourth of the peak after half a year or more.^26^ There have been reports that, even for COVID‐19, there are biological defense mechanisms that produce many new antibodies owing to the action of memory B cells following infection, even if the antibody concentration decreases. Furthermore, it has been reported that the number of memory T cells (helper T cells, killer T cells), which are useful in the biological defense mechanism, does not decrease. It is suggested that just the presence of memory cells can maintain immunity to a certain extent, even if the antibody concentration greatly decreases.^27^, ^28^, ^29^ Even then, the immunity decreases over time; Hence, the third booster dose (fourth dose in some countries) is being implemented worldwide.

Meanwhile, there are some countries where the second dose of the vaccine has not been sufficiently administered. Additionally, there is difficulty in conducting mass third‐dose vaccinations (boosters); therefore, it is important to set indicators of who should be given priority for additional vaccination in Japan. Our investigation showed that there was considerable variability between cases with regards to the neutralization of sVNT on day 180 (Figure 1B); therefore, it may be preferable to conduct neutralization assays to measure the Nabs titer and determine “the ability not only to recognize the virus but also to control infection.” There are many reports on whether to use neutralization assays to determine infection controllability following vaccination.^30^ However, further verification is needed to determine whether the same could be said for infection controllability following vaccination. However, the number of people infected with the Omicron variant is currently increasing despite the second dose of mRNA vaccinations. There is no indicator for “individual vaccine efficacy determination (virus recognition ability + infection control ability).” Given that we were able to investigate the correlation between sVNT and POCT‐sVNT from our neutralization test results (Figure 3B), POCT‐sVNT may be a candidate for this, and large‐scale prospective trials should be considered.

It is difficult to purchase expensive testing equipment and set up a mass sample analysis and testing systems in private medical institutions such as the facilities that participated in this study. In that sense, the existence of a point‐of‐care rapid neutralizing activity analysis kit that uses the immunochromatography method does not require a special environment. It can easily and rapidly conduct sVNT analysis. It might be thought that this would have a wide‐ranging social implementation, such as triage for the third dose of vaccination in clinical practice, triage for the extent of isolation restrictions after returning to Japan, indicator for staff reassignment (e.g., staff with reduced vaccine effects move to backyard shifts), and indicator for addition to vaccine passports in places such as restaurants, movie theaters, and events.

## 5 CONCLUSION

We used human serum samples from before and after mRNA vaccination to conduct a NAbs analysis and sVNT analysis over time. Specifically, our results showed large individual differences in reduction in neutralizing activity using conventional sVNT on day 180. In addition, we found that sVNT analysis might be helpful as an indicator for “possible virus recognizability and infection controllability” following vaccination. The more recently developed POCT‐sVNT has a high correlation with the conventional sVNT, which suggests the possibility of personal use.

## AUTHOR CONTRIBUTIONS

Watanabe Y and Degawa H designed and coordinated the study and performed the experiments, acquired, and analyzed data; Watanabe Y, Matsuba I, Watanabe K, Kunishima T, Takechi Y, Takuma T, Araki Y, Hirotsu N, Sakai H, Danno H, Fukuda M, Yamamoto H, Oikawa R, Itoh F, Oda I, Hatori Y, and Degawa H corrected samples and/or interpreted the data; Watanabe Y, Yamamoto H, Futagami S, Wada K, and Degawa H wrote or edited the manuscript; all authors approved the final version of the article.

## CONFLICT OF INTEREST

The authors state that they have no conflict of interest (COI).

## IRB APPROVAL CODE AND NAME OF THE INSTITUTION

The study was a prospective observational study, carried out by the opt‐in method of each institution, and approved by the ethics committees of the Kanagawa Medical Association (approval code: #R3‐0311). Informed consent for peripheral blood collection, Questionnaire, and testing was obtained from all participants. Details of our study design and results are uploaded to the Japan UMIN clinical trials registry website (registration number: UMIN000043851, scientific title: Short‐term safety of the BNT162b2 mRNA COVID‐19 vaccine) (https://center6.umin.ac.jp/cgi‐open‐bin/ctr/ctr_view.cgi?recptno=R000050049) and are available to share and download the files to all interested person, includes pdf format raw data, study protocol, statistical analysis plan, informed consent form, and clinical study report (from April 10, 2021).

## Data Availability

The data that provided the evidence for the study are available from the corresponding author upon reasonable request.
